# Advantages of a next generation sequencing targeted approach for the molecular diagnosis of retinoblastoma

**DOI:** 10.1186/s12885-015-1854-0

**Published:** 2015-11-04

**Authors:** Simona Grotta, Gemma D’Elia, Rossana Scavelli, Silvia Genovese, Cecilia Surace, Pietro Sirleto, Raffaele Cozza, Antonino Romanzo, Maria Antonietta De Ioris, Paola Valente, Anna Cristina Tomaiuolo, Francesca Romana Lepri, Tiziana Franchin, Laura Ciocca, Serena Russo, Franco Locatelli, Adriano Angioni

**Affiliations:** 1 Laboratory of Medical Genetics, Bambino Gesù Children’s Hospital, IRCCS, Piazza Sant’Onofrio 4, 00165 Rome, Italy; 2 Illumina, Inc, San Diego, CA 92122 USA; 3 Department of Pediatric Hematology-Oncology and Stem Cell Transplantation, Bambino Gesù Children’s Hospital, IRCCS, Piazza Sant’Onofrio 4, Rome, Italy; 4 Ophtalmology Unit, Bambino Gesù Children’s Hospital, IRCCS, Piazza Sant’Onofrio 4, Rome, Italy; 5 University of Pavia, Pavia, Italy; 6 Present address: S. Pietro Fatebenefratelli Hospital, UOSD Medical Genetics, Rome, Italy

**Keywords:** Retinoblastoma, Next-Generation Sequencing, *RB1* custom aCGH

## Abstract

**Background:**

Retinoblastoma (RB) is the most common malignant childhood tumor of the eye and results from inactivation of both alleles of the *RB1* gene. Nowadays RB genetic diagnosis requires classical chromosome investigations, Multiplex Ligation-dependent Probe Amplification analysis (MLPA) and Sanger sequencing. Nevertheless, these techniques show some limitations. We report our experience on a cohort of RB patients using a combined approach of Next-Generation Sequencing (NGS) and *RB1* custom array-Comparative Genomic Hybridization (aCGH).

**Methods:**

A total of 65 patients with retinoblastoma were studied: 29 cases of bilateral RB and 36 cases of unilateral RB. All patients were previously tested with conventional cytogenetics and MLPA techniques. Fifty-three samples were then analysed using NGS. Eleven cases were analysed by *RB1* custom aCGH. One last case was studied only by classic cytogenetics. Finally, it has been tested, in a lab sensitivity assay, the capability of NGS to detect artificial mosaicism series in previously recognized samples prepared at 3 different mosaicism frequencies: 10, 5, 1 %.

**Results:**

Of the 29 cases of bilateral RB, 28 resulted positive (96.5 %) to the genetic investigation: 22 point mutations and 6 genomic rearrangements (four intragenic and two macrodeletion). A novel germline intragenic duplication, from exon18 to exon 23, was identified in a proband with bilateral RB. Of the 36 available cases of unilateral RB, 8 patients resulted positive (22 %) to the genetic investigation: 3 patients showed point mutations while 5 carried large deletion. Finally, we successfully validated, in a lab sensitivity assay, the capability of NGS to accurately measure level of artificial mosaicism down to 1 %.

**Conclusions:**

NGS and *RB1*-custom aCGH have demonstrated to be an effective combined approach in order to optimize the overall diagnostic procedures of RB. Custom aCGH is able to accurately detect genomic rearrangements allowing the characterization of their extension. NGS is extremely accurate in detecting single nucleotide variants, relatively simple to perform, cost savings and efficient and has confirmed a high sensitivity and accuracy in identifying low levels of artificial mosaicisms.

## Background

Retinoblastoma (RB, OMIM:180,200) is the most common malignant childhood tumor of the eye with an estimated incidence between 1 in 16,000 and 1 in 18,000 live births [1, 2]. RB is the first disease for which a genetic etiology of cancer has been described [3] being caused by mutations in the first tumor suppressor gene identified (*RB1*, Genbank accession # L11910). Mutations in both alleles of the *RB1* gene are required for the development of this neoplasm [4], and, depending on the germ-line or somatic origin of the defect, a heritable or sporadic form can be distinguished. RB is unilateral in 60 % of cases and only 15 % of these are heritable [5]; in contrast, 40 % of retinoblastomas are bilateral with risk of transmission to the offspring. Heritable retinoblastoma constitutes a cancer predisposition syndrome [6]. *RB1* is located on chromosome 13 at band q14 and can be affected by a heterogeneous spectrum of genetic abnormalities, including chromosome translocation/deletion, genomic rearrangements, ranging from whole gene microdeletion to intragenic exons loss or duplication, and more than 900 different point mutations [7]. Mutational analysis is performed to search for the predisposing *RB1* gene mutation in peripheral blood of patients with RB, but the molecular diagnosis requires several technical approaches to cover the entire field of oncogenic *RB1* defects, frequently resulting in numerous, expensive and time consuming procedures. In particular, cytogenetic tools, such as classical chromosome investigations and Fluorescent In Situ Hybridization (FISH), in addition to Multiplex Ligation-dependent Probe Amplification (MLPA) technique, may account for detection of about 16 % of *RB1* abnormalities [8], while the remaining large amount of point mutations need to be investigated using sequencing analysis. Since the 1970s, Sanger sequencing has been recognized as the gold standard for mutation analysis in molecular diagnostics; however, its low-throughput, long turnaround time and overall cost [9] have called for new paradigms. Next Generation Sequencing (NGS) can massively sequence millions of DNA segments, promising low costs, increased workflow speed and enhanced sensitivity in mutation detection [9–11]. On the other hand, conventional and molecular cytogenetic analysis, have been replaced by modern high-throughput investigations, such as array Comparative Genomic Hybridization (aCGH), that can reveal and measure cryptic genomic imbalances. In addition, aCGH can be focused on specific DNA segments or genes maximizing the resolution *via* a customized process. Based on these observations, we have recruited a cohort of retinoblastoma patients we previously investigated with conventional cytogenetics and MLPA. Patients diagnosed with RB but negative to the above standard screening have been tested with NGS to assess its ability in identifying RB causative mutations. On the other hand, patients positive to standard screening have been further investigated with *RB1*-custom array CGH analysis to characterize the genomic rearrangements with a better resolution compared to the conventional techniques.

## Methods

### Patient recruitment

In this study we enrolled 65 patients affected by RB from the Department of Pediatric Hematology-Oncology and Stem Cell Transplantation of the Bambino Gesù Children’s Hospital in Rome. The study was approved by Ethical committee scientific board of Bambino Gesù Children’s Hospital and was conducted in accordance with the Helsinki Declaration. Blood samples were drawn from 64 patients after obtaining written informed consent from parents/guardians of affected children. Genomic DNA was extracted from peripheral blood with Qiagen columns (QIAamp DNA minikit; Qiagen, Hilden, Germany) according to the manufacturer’s instructions. Concentration and purity of DNA samples were quantified by ND-1000 spectrophotometer (NanoDrop; Thermo Scientific, Waltham, MA, USA). DNA samples were used either for NGS or aCGH technique. All 65 patients were previously tested with conventional cytogenetics and MLPA techniques. Fifty-three patients, resulted negative to the first screening, underwent molecular investigation. Eleven patients, where defects ranging from macroscopic deletions to intragenic rearrangements have been identified during the first study, were further characterized by *RB1* custom aCGH. Among these, one patient, positive to MLPA analysis resulted negative to aCGH. This patient was then further investigated by single exon conventional Sanger sequencing. As last, one more patient, positive to the cytogenetic analysis could not be further studied by aCGH as no DNA was available at the time of the test (Table 1).

**Table 1:** Cohort of patients enrolled in the study and techniques used for their characterization

Cohort	Cytogenetic - MLPA technique	Samples (technique)	Samples (RB)	# samples characterized by NGS or aCGH
65 patients	53 negatives	53 (NGS)	22 (BRB)	21
			31 (URB)	3
	12 positives	11 (aCGH)	6 (BRB)	5
			5 (URB)	5
		1 - no DNA available	1 (BRB)	-

### Targeted re-sequencing

Targeted resequencing was performed with a uniquely customized design: TruSeq® Custom Amplicon (Illumina, San Diego, CA) using the MiSeq® sequencing platform (Illumina). TruSeq Custom Amplicon (TSCA) is a fully integrated end-to-end amplicon sequencing solution, including online probe design and ordering through the Illumina website, assay, sequencing, automated data analysis and offline software for reviewing results. Online probe design was performed by entering into the Design Studio (DS) software (Illumina) the target genomic regions [12]. DesignStudio is a personalized, easy-to-use, web-based sequencing assay design tool that enables to move from project initiation to design, review, and ordering. DesignStudio provides dynamic feedback to optimize target region coverage, reducing the time required to design custom projects. Once the design is completed, a list of amplicons (short regions of DNA covering the full target region) is visualized and their quality is assessed on the basis of the predicted amplicon score provided by DS. The amplicon score is an estimate of the relative performance of a particular amplicon compared to all others in the pool. DesignStudio returns only candidate amplicons that are predicted to work well in the multiplex TruSeq Custom Amplicon assay. TSCA kit produces the required targeted amplicons with the necessary adapters and indices for sequencing on the MiSeq® system without any additional processing. Library preparation and sequencing runs have been performed according to the manufacturer’s procedure. Two different TSCA panel designs have been generated to investigate the same regions of interest for *RB1* gene: promoter, all coding regions, exon-intron boundaries, 5′UTR and 3′UTR of RB. A first panel of 43 amplicons, each of 250 bp was designed, with a total length of 5045 bp (Panel A). The total coverage obtained by DS across the entire region of interest was 97 % with amplicons showing scores in the range of 60–98 %. Amplicons with a score lower than 60 % were excluded from the TSCA panel (3 % of the entire region of interest). A second panel was designed with amplicons of 425 bp in length for a total of 36 amplicons (Panel B). In this case, the predicted coverage of the full region of interest was 100 % with amplicons showing scores in the range of 60–98 %. Of the 53 patients studied with NGS, 48 patients were analyzed using panel A while 5 patients were analyzed using Panel B.

### Mosaicism detection rate assessment

To test the detection rate for mosaic mutations using the MiSeq, three different types of previously recognized mutations of RB patients, a substitution, an insertion and a double deletion, were diluted at different concentrations. DNA from normal individuals was mixed with the mutated DNA to obtain a final dilution of 10, 5 and 1 %. For this test all libraries were prepared using the TSCA Panel B. To compare the most appropriate protocol in terms of coverage required to discriminate a certain mosaicism frequency, these samples were sequenced at two different coverage levels: low coverage (600x) and high coverage (9000x).

### Data analysis

The MiSeq® system provides fully integrated on-instrument data-analysis software. The MiSeq Reporter software performs secondary analysis on the base calls and quality scores generated by Real Time Analysis (RTA) during the sequencing run. The type of analysis performed is based on the analysis workflow selected. The TruSeq Amplicon workflow evaluates short regions of amplified DNA, or amplicons, for variants. The TruSeq Amplicon workflow performs demultiplexing of indexed reads, generates FASTQ files, aligns reads to a reference, identifies variants, and writes output files to the Alignment folder. SNPs and short indels are identified using the Genome Analysis Toolkit (GATK). GATK calls raw variants for each sample, analyzes variants against known variants, and then calculates a false discovery rate for each variant. Each single variant has been evaluated for the coverage and the Qscore, and visualized *via* Amplicon Viewer (AV) and Integrative Genome Viewer (IGV) software [13, 14]. The Qscore is the prediction of the probability of an erroneous base call, in particular, a value of Q30 represents the probability to call an erroneous base out of 1000, reflecting an accuracy of the sequenced base of 99.9 %. All detected variants have been filtered based on their Qscore: only variants showing Qscore > 30 have been considered in this study. Coverage for a defined amplicon is the average number of sequencing reads representing a given nucleotide in that amplicon. All mutations identified by Miseq Reporter were validated by Sanger sequencing using standard protocols.

### *RB1* custom array CGH

Array-CGH was carried out using a 60-mer oligonucleotide-based microarray platform that allows molecular profiling of genomic aberrations with an overall median probe spatial resolution of 41 kb (60 K) (Agilent Technologies Array-CGH Kits, Santa Clara, CA) with an increased resolution of 1000 times in the customized region (88 bp median overall probe spacing) containing *RB1*. The design of the custom array slide was made using the Agilent website dedicated to this purpose [15]. In order to customize *RB1*, i.e., to get the maximum probe coverage of all the exonic and intronic regions of this gene and its 5′ (1000 bp) and 3′ (500 bp) segments, we chose all the probes available from Agilent (2046). Human genomic DNA was used as reference DNA. Aliquots of 350 ng of DNA from samples were fragmented with heat for 40 minutes at 99 °C. Then, each sample was labeled by random priming (Agilent Technologies) for 2 hours at 37 °C and 10 minutes at 65 °C using Cy5-dUTP for patient DNAs and Cy3-dUTP for reference DNAs. Labeled products were cleaned-up with SureTag DNA Labeling Kit Purification Columns (Agilent Technologies). After probe denaturation for 3 minutes and 30 seconds at 94 °C and pre-annealing with 2 μg of Cot-1 DNA for 30 minutes at 37 °C, hybridization was performed at 65 °C with rotation for 24 hours. After washing steps, following the manufacturer’s instructions, the arrays were analyzed using the Agilent scanner G2505C and Feature Extraction software v.10.7. A graphical overview of the results was obtained using Agilent Genomic Workbench v.7.0.

Copy number variations (CNVs) were identified with the ADM2 (Aberration Detection Method) algorithm and filtered consulting the Database of Genomic Variants [16].

## Results

Of the 65 patients, 64 were investigated either with NGS or aCGH. Fifty-three patients were analyzed with NGS: 22 were diagnosed with bilateral RB (BRB), while 31 with the unilateral form (URB). Indeed, 11 patients were studied with custom aCGH: 6 diagnosed with BRB and 5 with URB.

One last BRB patient, missing DNA for further investigation by aCGH, was analyzed by classic cytogenetics and showed a large deletion higher than 10 Mb.

### NGS

Fifty-three patients were analyzed with NGS in two different sequencing runs. Sequencing data generated were evaluated on the basis of the Qscore and coverage. In the case of mosaicism experiments, variant frequency was also evaluated. As predicted by DS coverage indication, Panel A confirmed coverage of 97 % of the full target region for all 48 patients studied in this first sequencing run. Exon 2 was only partially sequenced, while exons 14 and 20 were not sequenced at all. To achieve a full coverage of the target region, the reported exons had to be investigated by conventional Sanger sequencing. In the second sequencing run, where Panel B was used, the full target region (coding regions, promoter and splicing junctions) was completely sequenced as predicted by DS (100 %). In this second case, Sanger sequencing was carried-out only to confirm previously recorded mutations. The mean coverage achieved for each sample was 1196 for Panel A and 1309 for Panel B. All detected variants showed a mean coverage of 592 and a mean Qscore of 39 (99.87 % accuracy). An example of performance of Panel B is reported in Table 2. All but one of the 22 BRB patients have been found mutated with NGS. The patient that did not show any mutation was further analyzed with conventional Sanger sequencing confirming the absence of any mutation.

**Table 2:** Coverage level through the target region for patient ID 24 (library preparation performed with Panel B)

Exon	Amplicon start	Amplicon end	Coverage	Exon	Amplicon start	Amplicon end	Coverage
5’UTR+1	48877740	48878189	320	17	48955328	48955770	970
	48878120	48878544	499	18	49027054	49027468	1800
2	48881319	48881718	750	19	49030255	49030669	635
3	48916666	48917104	1500	20	49033602	49034002	2300
4	48919157	48919573	840		49033928	49034359	3000
5	48921888	48922320	1100	21	49037776	49038175	349
6	48923035	4982348	450	22	49038987	49039397	1300
7	48934082	48934522	2100	23	49039325	49039761	600
8	48936850	18937296	800	24	49047419	49047829	300
9	48938842	48939254	3150	25	49050772	49051184	2900
10	48971551	48941965	3692	26	49051422	49051822	296
11	48942426	48942874	65	27	49053981	49054380	5000
	48972798	48943224	135	3’UTR	49054269	49054693	1957
12	48947438	48947854	1371	3’UTR	49051617	49055024	2607
13	48950985	48951409	1500	3’UTR	49054949	49055395	1321
14	48953395	48953828	659	3’UTR	49055319	49055740	2847
	48953757	48954206	456	3’UTR	49055661	49056085	428
15-16	48954127	48954562	431	3’UTR	49056009	49056429	2458

Of the 21 identified pathogenic mutations, one was associated with a rare case of trilateral retinoblastoma. As regards the 31 URB group, variants have been detected in 3 patients. The features and assortment of all the mutations found are summarized in Table 3.

**Table 3:** List of all mutations identified either by NGS or Sanger sequencing

ID	Laterality	Exon intron	Coordinate	Type	Allele	Q	Coverage	Variant frequency	Mutation	Protein	References
1	TRB	2	48881497	Deletion	het	40	446	0.51	c.220_221delGC	p.Ala74fs35X	New
2	BRB	2	48881523	SNP	het	38	167	0.43	c.245C>A	p.Ser82X	[26]
3	BRB	2	48881542	Deletion	het	/	/	/	c.264delG	Altered splicing	New
4	BRB	3	48916744	Insertion	het	39	1463	0.5	c.274insT	p.Ile92fs109X	New
5	BRB	3	48916831	SNP	het	38	1409	0.38	c.316C>T	p.GLn121X	New
6	BRB	3	48916839	Deletion	het	37	599	0.51	c.369delAT	p.Asn123fs129X	New
7	BRB	8	48937069	Deletion	het	40	450	0.51	c.837_841delGAACA	p.Glu280del_His281X	New
		8	48937075	Deletion	het	40	453	0.51	c.843delG		
8	BRB	8	48937095	SNP	het	38	250	0.48	c.861+2T>C	Altered splicing	[29]
9	BRB	10	48941648	SNP	het	40	1250	0.49	c.958C>T	p.Arge320X	[29]
10	BRB	11	489742685	SNP	het	39	421	0.52	c.1072C>T	p.Arg358X	[30]
11	URB	IVS-12	48947629	SNP	het	39	565	0.52	c.1215+1G>T	Altered splicing	COSMIC-COSMIC29786
12^a^	BRB	IVS-13	48953729	SNP	het	/	/	/	c.1333-1G>A	Altered splicing	[31]
13	BRB	15	48954198	SNP	het	37	231	0.47	c.1399C>T	p.Arg467X	[32]
14	BRB	15	48954198	SNP	het	36	343	0.46	c.1399C>T	p.Arg467X	[32]
15	BRB	18	49027139	SNP	het	40	1073	0.49	c.1706T>A	p.Leu569X	New
16	BRB	IVS-19	49033822	SNP	het	38	626	0.45	c.1961-2A>G	Skip exone 19	[33]
17	BRB	IVS-19	49030486	SNP	het	39	227	0.45	c.1960+1G>A	Altered splicing	[34]
18	BRB	20	49033935	Deletion	het	39	305	0.47	c.2073delG	p.Glu691fs695X	New
19	BRB	IVS-21	49037976	SNP	het	40	593	0.54	c.2211+5G>A	Altered splicing	[35]
20	BRB	22	49039209	SNP	het	40	452	0.45	c.2287A>T	p.Arg763X	New
21	BRB	23	49039374	SNP	het	40	234	0.51	c.2359C>T	p.Arg787X	[29]
22	URB	23	49039374	SNP	het	39	248	0.51	c.2359C>T	p.Arg787X	[29]
23	BRB	23	49039444	Insertion	het	39	860	0.49	c.2429insGTTC	p.Lys810fs815X	New
24	URB	25	49050852	SNP	het	40	960	0.44	c.2536C>T	p.Gln846X	[26]
25^b^	BRB	7	48934197	Deletion	het	/	/	/	c.652delT	p.Leu218X	New

^a^Patients with mutation detected by Sanger as integration of uncovered regions from Panel A

^b^Patient negative to array-CGH, re-analysed by Sanger sequencing on the same exon previously identified positive by MLPA

### CGH array

**Fig. 1:**
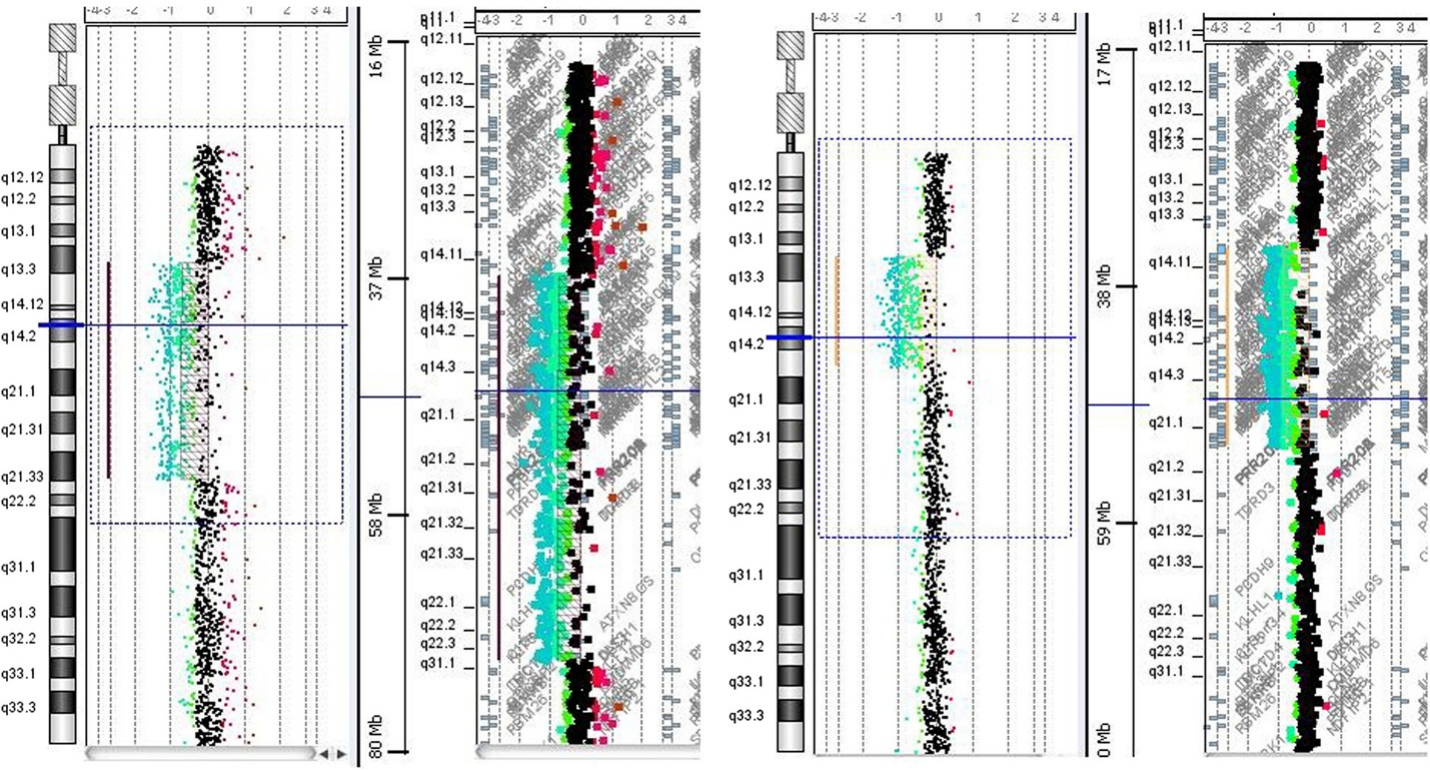
aCGH profiles of  large deletions in patients with URB and TRB

Eleven patients, showing genomic abnormalities, were properly characterized in length and position by *RB1* custom aCGH analysis (Fig. 1). All five patients with URB showed only large deletion while in five out six BRB patients were found three small intragenic deletions, one extended intragenic duplication, unexpectedly presenting syndromic features, and one large deletion. The sample found negative by a-CGH was further analysed by conventional Sanger sequencing focusing on the same exon recognised as deleted by MLPA. Sanger sequencing confirmed the presence of a point mutation. Genomic rearrangements and their characteristics are reported in Table 4. In conclusion, the overall number of RB patients with point mutations or genomic rearrangements identified by either NGS or aCGH was 28 out of a total of 29 BRB patients (96.5 %) and 8 out of 36 URB patients (22 %).

**Table 4:** List of all genomic rearrangements identified by aCGH or karyotype analysis

ID	Laterality	Cytogenetics	MLPA	a-CGH	Size
26	TRB	46,XX,del(13)(q14q22)	Del whole gene	arr 13q13.3q14.3(35,876,405-53,551,359)x1	17.7 Mb
27^a^	BRB	46,XY,del(13)(q13q14)	/	/	>10 Mb
28	BRB	46, XY	Dup exon 18 to 23	arr 13q14.2(48,973,699-49,039,548)x3	65.8 Kb
29	BRB	46,XX	Del exon 1a-1b	arr 13q14.2(48,877,905-48,878,660)x1	755 bp
30	BRB	46,XX	Del exon 17	arr 13q14.2(48,954,774-48,955,679)x1	905 bp
31	BRB	46, XY	Del exon 3 to 6	arr 13q14.2(48,902,145-48,923,382)x1	21.2 Kb
25^b^	BRB	46, XX	Del exon 7	Negative	/
32	URB	46,XY,del(13)(q14q22)	Del whole gene	arr 13q13.3q21.33(38,225,360-72,646,762)x1	34.5 Mb
33	URB	46,XY,del(13)(q14q22)	Del whole gene	arr 13q14.11q21.33(43,793,461-69,444,583)x1	25.7 Mb
34	URB	46, XY	Del whole gene	arr 13q14.11q14.2(43,793,461-49,523,881)x1	5.7 Mb
35	URB	46, XY	Del whole gene	arr 13q14.2(47,343,288-49,047,329)x1	1.74 Mb
36	URB	46, XY	Del whole gene	arr 13q14.2(47,657,454-49,309,890)x1	1.65 Mb

^a^Genomic rearrangements detected by karyotype analysis (DNA not available)

^b^Patient found positive by MLPA, negative by array-CGH, re-analyzed by Sanger method focused on the same exon previously recognized by MLPA as delete

### Mosaicism detection rate assessment

Dedicated experiments were carried out to investigate the lowest limit of the NGS method in detecting targeted mutational mosaicism rate. Results are summarized in Table 5. All variants were correctly identified at each mosaicism frequency for both sequencing runs (600x and 9000x). Only small differences from the expected frequency have been observed and this could be probably related to the variability associated to the handling, pipetting and preparation of the dilutions.

**Table 5:** Artificial mosaicism detection frequencies obtained with NGS experiment (Low coverage sequencing run and high coverage sequencing run)

ID	Mutation type	Diluiton (%)	Variant Coverage	Variant call (%)	False positive (%)	Variant Coverage	Variant call (%)	False Positive (%)
22	c.2359C > T	10.00 %	35	7.00 %	Not calculated	571	6.00 %	Not calculated
		5.00 %	24	5.00 %	Not calculated	220	4.00 %	Not calculated
		1.00 %	7	2.00 %	0.00 %	239	2.97 %	0.01 %
ID	Mutation type	Diluiton (%)	Variant Coverage	Variant call (%)	Error insertions (%)	Variant Coverage	Variant call (%)	Error insertions (%)
4	c.274insT	10.00 %	163	9.00 %	Not calculated	2946	10.4 %	Not calculated
		5.00 %	60	6.25 %	Not calculated	707	5.8 %	Not calculated
		1.00 %	1	2.00 %	0.00 %	9	1.60 %	0.00 %
ID	Mutation type	Diluiton (%)	Variant Coverage	Variant call (%)	Error deletions (%)	Variant Coverage	Variant call (%)	Error deletions (%)
7	c.837_841delGAACA	10.00 %	35	4.76 %	Not calculated	503	6.1 %	Not calculated
		5.00 %	30	3.6 %	Not calculated	298	2.97 %	Not calculated
		1.00 %	6	0.76 %	0.00 %	51	0.6 %	0.02 %
	c.843delG	10.00 %	37	5.00%	Not calculated	508	6.1 %	Not calculated
		5.00 %	31	3.7 %	Not calculated	299	2.98 %	Not calculated
		1.00 %	6	0.76 %	0.00 %	49	0.6 %	0.00 %

Variant call % and error % here reported have been filtered for Qscore > 30

For the 1 % mosaicism frequency, it has been evaluated the frequency of false positive calls in terms of erroneously called bases in the target site. In details, as regards all three types of variants studied, the frequency of false positive events has always been between 0 and 0.02 % for both sequencing runs. In particular, for the high coverage sequencing run, the false positive events never exceeded 0.02 %.

### Availability of supporting data

The microarray and sequencing raw data are available in the ArrayExpress database (www.ebi.ac.uk/arrayexpress) under accession numbers respectively E-MTAB-3492 and E-M-TAB-3515.

## Discussion

The molecular diagnosis of RB is a complex and articulate process that still represents an exciting challenge. Many resources and skills need to be involved to obtain satisfactory results. High-throughput technologies can actually offer new opportunities in relation to the amount of genes potentially analyzed, the number of samples examined and the quality of results. NGS is an innovative technology that is able to massive-parallel sequence millions of DNA segments with high definition capability. It has a wide diffusion in many fields of biomedical research, but diagnostic applications for genetic diseases are still in progress. We report our experience on a cohort of RB patients using a NGS approach on the Illumina MiSeq platform. The experiments required different timelines. The design of the target regions of *RB1*, carried out using DS, was performed in few hours. The preparation of the genomic library using the TSCA Illumina kit, was completed in two working days. One or two days were spent to run the samples on the MiSeq (48 samples were run all together in a first sequencing run using Panel A and the remaining 5 were run on a second experiment using Panel B). Few more days were required for results interpretation of the 53 RB patients using MiSeqReporter, AV and IGV2.3 software. Furthermore, all mutations identified by Miseq Reporter, were validated by Sanger sequencing using standard protocols. Of the two panels designed, Panel B has allowed to reach the full coverage of the target region, making the standard Sanger sequencing only a tool for confirming all detected variants. It was also calculated that the cost of NGS analysis for the entire *RB1* gene, considering comparable devices cost, reagents expenses, operator’s worktime, would be 7 times less than the cost of a protocol entirely based on Sanger sequencing, allowing a strong decrease in costs and a large increase in the number of samples processed for each experiment [9, 17, 18].

NGS has allowed identifying all variants found in patients with BRB except one sample in which the variant was identified neither by Sanger nor by NGS sequencing. In this case we can speculate that the variant may be located outside the region under investigation. In fact, literature data show that 5 % of cases with bilateral involvement may have translocations, deep intronic splice site mutations, or low-level mosaic mutations, which may or may not be germline [8]. Twenty-four mutations were identified in the patients with RB: twelve nonsense, five frameshift and seven splice site mutations. As expected, eleven out of the twenty-four mutations found were newly discovered mutations, never reported before. Among these a rare case of trilateral RB with a new frameshift mutation in exon 2 was identified, differently from the current data reporting macroscopic deletions as the most frequent defects in this unusual disease [19–22]. The nonsense mutation *p.Arg787X* was a known sequence variation found in the group of URB. The carrier was a female presenting, at the age of 17 months, with a left eye RB with loco-regional metastasis also involving lymphnodes and bone marrow. She was eye enucleated and treated with conventional and high-dose chemotherapy, followed by autologous bone marrow transplantation and radiotherapy. To date, she is alive and in good clinical conditions. *p.Arg787X* is a recurrent mutation commonly found in BRB as germline sequence variation, while in URB is more frequent as somatic mutation. Only four cases of URB carrying this germline mutation have been reported [23] including a patient with metastatic presentation [24]. These findings suggest that the phenotypic expression of *p.Arg787X* may reflect the variable penetrance of this defect, leading to the different pictures of the disease. Among the genomic abnormalities identified with *RB1*-custom aCGH method, four intragenic rearrangements and six large deletions involving genes adjacent to *RB1* were revealed. Interestingly, the patients belonging to the first group had BRB, while the patients of the second group had mainly URB. These data fortify the hypothesis that deletion of genes essential for cell survival, adjacent to *RB1*, may cause less invasive tumors and, therefore, result in a higher frequency of unilateral disease [25, 26]. Patients with deletions greater than 5.7 Mb showed syndromic features with variable degree of intellectual disability ranging from moderate to severe. Patients with deletions smaller than 1.74 Mb had only RB. An unexpected exception was the case of the proband with BRB carrying an intragenic duplication from exon 18 to 23, lasting about 66 Kb. This patient presented with a clinical syndromic picture, characterized by macrosomia, nystagmus of the eye, macrocephaly and macroglossia evocative of the Beckwith-Wiedeman Syndrome. Molecular investigations revealed a normal methylation status and absence of microdeletions at the locus 11p15.5. Array-CGH did not show any genomic imbalances. In this cohort, 8 out 36 URB patients resulted positive either to NGS or aCGH, corresponding to a 22 % frequency. URB mutations are in fact infrequently found (15 %) in blood circulating cells in relationship to the known prevalence of somatic mutation in the target tissue. The 22 % frequency here reported is slightly different from what is reported in literature, however, the small number of patients in this cohort is not enough to establish a significant frequency reference.

Mutational mosaicism is an exciting challenge regarding molecular diagnostics as well as it is important in the genetic counseling setting. Low levels of mutational mosaicism have been identified in probands with bilateral disease and in individuals with unilateral disease who have affected children inheriting the mutation [8, 27]. Conventional investigations are unable to routinely detect low-rate mutated cells: currently, Sanger sequencing is able to disclose mosaicism only for rates above 20 %. Targeted mutation analysis is useful to study mosaic recurrent mutations in blood and can detect DNA variations below the limit of standard Sanger sequence analysis. This type of analysis, based on Allele Specific PCR (AS-PCR), however, investigates, only a limited number of recurrent point mutations [26]. A more recent study demonstrated that, using a deep semiconductor sequencing approach (Ion Torrent, Life Technology), the detection rate of targeted mutational mosaics can be revealed at a frequency down to 5 % [28]. In our study the capability of NGS in detecting low mosaicism frequency has been tested. Due to the absence of patients with RB1 mosaicism, three previously recognized samples, carriers of single-base substitution, single-base insertion and a complex rearrangement involving five-base and one-base double deletion respectively, were diluted with normal DNA at different concentration (10, 5, 1 %) and tested by NGS with MiSeq platform. As reported, all three mutations have been correctly detected at each different frequency for both coverage levels, independently of the variant type. When leading studies aimed at identifying low mosaicism frequencies, the major difficulty lies in accurately discriminating between a somatic variant and a false positive episode. Based on this, for all three studied mutations, it has been evaluated the frequency of false positive calls measured as the percentage of erroneously called bases at the target site. As shown, for all three types of variants studied, the frequency of false positive events has always been between 0 and 0.02 % for both sequencing runs. In particular, for the high coverage sequencing run, the false positive events never exceeded 0.02 %, far below the 1 % mosaicism variant frequency detected. This achievement, accompanied by a good coverage of the region of interest can accurately detect low mosaicism frequencies in biological samples, providing a reliable and sensitive method of screening. Validation experiments on mosaic biological samples are currently in progress.

## Conclusions

NGS and *RB1*-custom array CGH demonstrated to be an effective association in order to optimize the overall diagnostic procedures of RB. The major advantages provided by NGS are the high performance capacity and the elevated accuracy in the data generated. Quality and quantity of the results acquired in months of traditional work, are achieved in a single experiment and this contributes to an extraordinary abatement of the global cost.

NGS has also allowed the identification of artificial mosaicism frequencies down to 1 %, providing consistent data, high accuracy and extremely low frequency of false positive events (0.02 %). The possibility to analyze hundreds of samples per experiment and to sequence different genes simultaneously makes NGS a powerful and innovative tool for a modern approach to study rare diseases.

## Abbreviations

aCGH: Array Comparative Genomic Hybridization
ADM2: Aberration Detection Method
AS-PCR: Allele Specific PCR
AV: Amplicon Viewer
BRB: Bilateral retinoblastoma
CNVs: Copy number variations
DS: Design Studio
FISH: Fluorescent In Situ Hybridization
GATK: Genome Analysis Toolkit
IGV: Integrative Genome Viewer
MLPA: Multiplex Ligation-Dependent Probe Amplification
NGS: Next Generation Sequencing
Qscore: Quality score
RB: Retinoblastoma
RTA: Real time analysis software
TSCA: TruSeq Custom Amplicon
URB: Unilateral retinoblastoma
VCF: Variant call file
